# Targeting ornithine decarboxylase reverses the LIN28/Let-7 axis and inhibits glycolytic metabolism in neuroblastoma

**DOI:** 10.18632/oncotarget.2768

**Published:** 2014-11-15

**Authors:** Ann M. Lozier, Maria E. Rich, Anissa Pedersen Grawe, Anderson S. Peck, Ping Zhao, Anthony Ting-Tung Chang, Jeffrey P. Bond, Giselle Saulnier Sholler

**Affiliations:** ^1^ Pediatric Oncology Translational Research Program, Helen DeVos Children's Hospital, Grand Rapids, MI, USA; ^2^ Small Animal Imaging Facility, Van Andel Institute, Grand Rapids, MI, USA; ^3^ University of Vermont, Michigan State University, Grand Rapids, MI, USA; ^4^ College of Human Medicine, Michigan State University, Grand Rapids, MI, USA

**Keywords:** neuroblastoma, DFMO, LIN28B, glycolytic metabolism, stem cells

## Abstract

*LIN28* has emerged as an oncogenic driver in a number of cancers, including neuroblastoma (NB). Overexpression of *LIN28* correlates with poor outcome in NB, therefore drugs that impact the LIN28/Let-7 pathway could be beneficial in treating NB patients. The LIN28/Let-7 pathway affects many cellular processes including the regulation of cancer stem cells and glycolytic metabolism. Polyamines, regulated by ornithine decarboxylase (ODC) modulate eIF-5A which is a direct regulator of the LIN28/Let-7 axis. We propose that therapy inhibiting ODC will restore balance to the LIN28/Let-7 axis, suppress glycolytic metabolism, and decrease MYCN protein expression in NB. Difluoromethylornithine (DFMO) is an inhibitor of ODC in clinical trials for children with NB. *In vitro* experiments using NB cell lines, BE(2)-C, SMS-KCNR, and CHLA90 show that DFMO treatment reduced LIN28B and MYCN protein levels and increased Let-7 miRNA and decreased neurosphere formation. Glycolytic metabolic activity decreased with DFMO treatment *in vivo.* Additionally, sensitivity to DFMO treatment correlated with *LIN28B* overexpression (BE(2)-C>SMS-KCNR>CHLA90). This is the first study to demonstrate that DFMO treatment restores balance to the LIN28/Let-7 axis and inhibits glycolytic metabolism and neurosphere formation in NB and that PET scans may be a meaningful imaging tool to evaluate the therapeutic effects of DFMO treatment.

## INTRODUCTION

Neuroblastoma (NB) is a pediatric cancer that is generally diagnosed in children before the age of five [1]. NB arises from cells of the sympathetic nervous tissue and primary tumors are typically found in the adrenal gland, but can also be found along sympathetic nerves in the neck, chest, abdomen, and pelvis [1]. NB tumors are highly invasive and often metastasize to secondary sites including bone, lymph nodes, liver, and bone marrow [1]. High risk NB patients are characterized by *MYCN* amplification, age greater than 18 months, unfavorable histology, and aggressive metastatic disease [2, 3]. With multimodal treatments these patients can achieve remission but many who go into remission will relapse, at which point long term survival is less than 10% [1].

Studies have shown that polyamines play an important role in many cancers including NB [4-6]. The rate limiting enzyme of polyamine biosynthesis is Ornithine decarboxylase (ODC). The *ODC* gene is found upstream of *MYCN* [7] and since *MYCN* amplification is seen in 30% of neuroblastoma, *ODC* overexpression is common in NB patients and correlates with poor outcomes [1]. ODC's specific function is to convert the polyamine precursor ornithine into the polyamine putrescine. Putrescine is then converted to the polyamines spermidine and spermine in the polyamine metabolism pathway. Little is known about the specific functions of polyamines; however, preclinical and early studies targeting polyamines in cancer have yielded promising results [4, 8, 9].

One specific function of spermidine has been identified. Spermidine is a substrate needed for post-transcriptional modification of the translation initiation factor eIF-5A, a process called hypusination [10-12]. Recent studies show that eIF-5A is a direct regulator of the LIN28/Let-7 pathway [13, 14], which is important in a number of cancers, including NB, and was recently identified as a pathway of particular importance in tumor initiating cells [15]. LIN28 is a miRNA binding protein that is highly expressed in undifferentiated cells and has reported oncogenic properties [16]. Let-7 is a miRNA that acts as a tumor suppressor by inhibiting oncogene transcription [17-19]. LIN28 and Let-7 are highly regulated throughout development and interact via a double negative feedback loop in which LIN28 inhibits Let-7 and vice versa [16]. Recent studies have shown that high *LIN28B* expression is associated with worse survival outcomes in NB patients and that deletion of *LIN28B* significantly reduces NB cell growth [20]. Additionally, the LIN28 and Let-7 are both linked to MYCN protein expression, therefore the LIN28/Let-7 pathway may be particularly important in high risk, *MYCN*-amplified NB patients [21].

**Figure 1:**
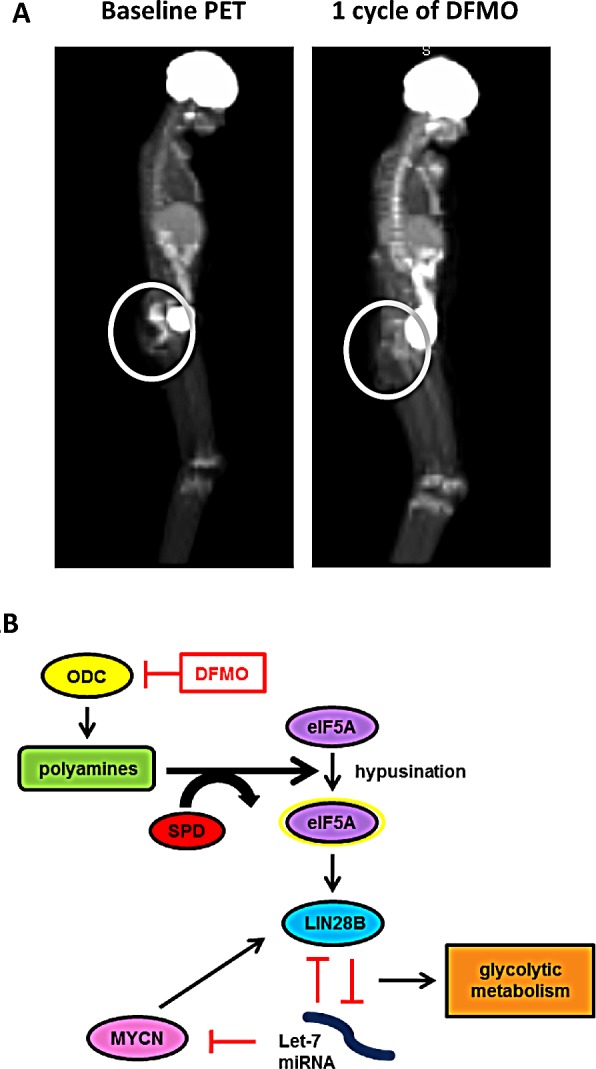
A) Patient PET scans Patient treated on Phase I study of DFMO tablets with 1000mg/m2 twice daily shows reduced tumor metabolic activity after one cycle of DFMO treatment, indicated by decreased ^18^F-FDG uptake in tumors. After starting DFMO treatment, the patient's tumor was still present, although showed negative PET avidity. Decreased PET activity is an indication of reduced glycolytic metabolism. B) Proposed pathway of DFMO effects on LIN28/Let-7 axis and glycolytic metabolism in NB. DFMO reversibly inhibits ODC, leading to reduced hypusination of eIF5A and a reversal of the LIN28/Let-7 axis. Changes in LIN28/Let-7 axis likely contribute to reduced glycolytic metabolic activity seen in NB cells treated with DFMO.

A recent study showed that the LIN28/Let-7 axis directly regulates glycolytic metabolism [22], a process that is important in both cancer cells and stem cells. In this study, LIN28 enhanced glucose uptake via an increase in insulin-PI3K-mTOR signaling, which regulates glycolytic metabolism. Let-7 was shown to suppress this pathway, thereby reducing glucose uptake and downregulating glycolytic metabolism [22]. Elevated glycolytic metabolism is a characteristic common in many cancers, including NB [23, 24]. Thus, targeting the LIN28/Let-7 axis may be a beneficial method for decreasing tumor metabolic activity in these cancers (Figure 1).

Difluoromethylornithine (DFMO) is a drug that inhibits polyamine biosynthesis, thus impacting the LIN28/Let-7 axis. DFMO was originally developed over 30 years ago as chemotherapeutic agent and has since been widely used as a treatment for parasitic diseases. Although early clinical studies with DFMO were disappointing, more recent trials using DFMO in combination with other chemotherapies or as a single chemopreventative agent have shown promising results [9, 25-29]. DFMO works by irreversibly binding to and inhibiting ODC. When ODC is inactive, ornithine cannot be converted to putrescine, which hinders the downstream production of spermidine and spermine and prevents the hypusination of eIF-5A [12]. Studies have shown that DFMO treatment effectively reduces polyamine levels of NB cells *in vitro* [4], and clinical trials are currently underway using DFMO to treat cancer, including NB.

Recent anecdotal clinical cases have shown that DFMO treatment reduces glycolytic metabolic activity of tumors in patients (Figure 1). In three separate cases, NB patients enrolled in a Phase I DFMO clinical trial had reduced tumor metabolic activity as measured by Positron Emission Tomography (PET) scans after starting DFMO treatment and exhibited prolonged stabilization of disease [9]. In light of these clinical observations and recent studies linking polyamines to the LIN28/Let-7 axis [13] and LIN28/Let-7 to glycolytic metabolism [22], we hypothesize that treatment with DFMO will restore balance to the LIN28/Let-7 axis, thereby reducing glycolytic metabolism in NB. We also believe that, due to the direct interaction between LIN28 and MYCN [21], NB cell lines that have high *LIN28B* and *MYCN* expression will be more sensitive to DFMO treatment.

## RESULTS

### *LIN28B* and *MYCN* genes are variably expressed in NB cell lines

Microarray analysis was used to assess relative levels of LIN28B and MYCN RNA expression in three NB cell lines: BE(2)-C, SMS-KCNR, and CHLA90. Expression levels were compared to a normal human tissue control reference set and all p-values are adjusted p-values, with a threshold of less than 0.05. Fold change in expression for each cell line is shown in Table 1. *LIN28B* and *MYCN* gene expression varied in the three NB cell lines. BE(2)-C cells highly expressed both *LIN28B* (83-fold increase, *p* = 1.1E-14) and *MYCN* (399-fold increase, *p* = 4.3E-19) relative to SMS-KCNR and CHLA90 cells, SMS-KCNR cells exhibited moderate expression of *LIN28B* (14-fold increase, *p* = 4.4E-8) relative to BE(2)-C cells and high expression of *MYCN* (426-fold increase, *p* = 3.9E-19), and CHLA90 cells had low expression of both *LIN28B* (6.4-fold increase, *p* = 5.1E-5) and *MYCN* (5.1 fold increase, *p* = 3.4E-4) relative to the BE(2)-C and SMS-KCNR cell lines.

**Table 1:** *LIN28B* and *MYCN* gene expression in three NB cell lines All fold change values were calculated compared to the whole body reference set. BE(2)-C cells exhibited high *LIN28B* and *MYCN* expression. SMS-KCNR cells had moderate *LIN28B* expression and high *MYCN* expression. CHLA90 cells exhibited low gene expression for both *LIN28B* and *MYCN* relative to BE(2)-C and SMS-KCNR cell lines

	*LIN28B* Fold Change	*MYCN* Fold Change
**BE(2)-C**	82.6 (*p* = 1.1E-14)	398.6 (*p* = 4.3E-19)
**SMS-KCNR**	14.3 (*p* = 4.4E-8)	425.5 (*p* = 3.9E-19)
**CHLA90**	6.4 (*p* = 5.1E-5)	5.1 (*p* = 3.4E-4)

### Cell lines with high *LIN28B/MYCN* gene expression are more sensitive to DFMO treatment

**Figure 2:**
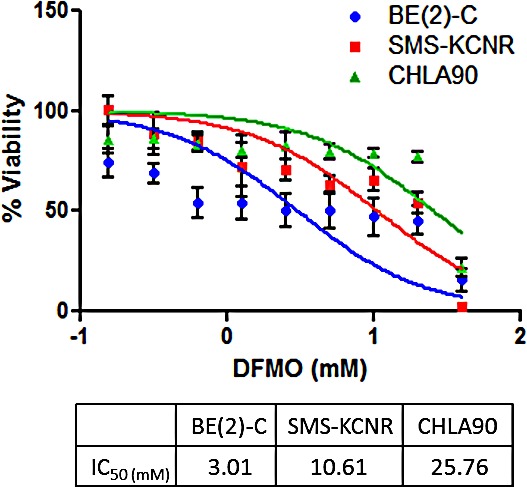
Sensitivity to DFMO treatment in three NB cell lines DFMO IC_50_ values were calculated after 72 hours of treatment; results are the average of 4 independent experiments. BE(2)-C cells were most sensitive to DFMO treatment with an IC_50_ of 3.0 mM, SMS-KCNR cells exhibited moderate sensitivity with an IC_50_ of 10.6 mM, and CHLA90 cells were the least sensitive to DFMO treatment with an IC_50_ of 25.8 mM.

**Figure 3:**
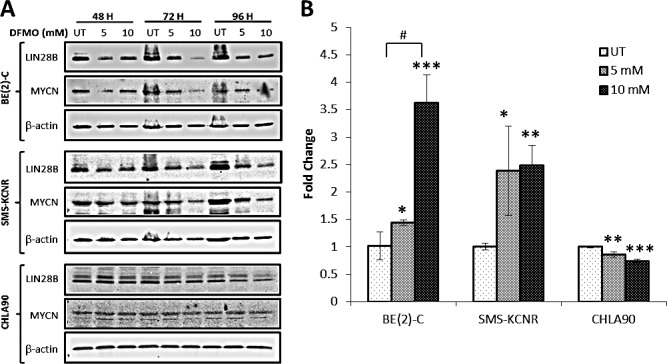
DFMO treatment reverses the LIN28B/Let-7 axis in NB A) Western blot analysis of LIN28B and MYCN protein levels in cells treated with DFMO. Cells were treated with 5 mM or 10 mM DFMO for 48-96 hours. LIN28B and MYCN protein expression decreased with DFMO treatment in BE(2)-C and SMS-KCNR cells at all three timepoints. LIN28B protein expression decreased in CHLA90 cells with DFMO treatment, but MYCN protein expression did not change. B) qRT-PCR analysis of Let-7 miRNA expression in NB cells after 6 hours of treatment with DFMO. qRT-PCR data is presented as fold change compared to untreated. BE(2)-C and SMS-KCNR Let-7 miRNA expression was significantly increased in cells treated with DFMO. Let-7 miRNA expression significantly decreased in CHLA90 cells treated with DFMO (*p < 0.05, **p < 0.01, ***p < 0.001 relative to untreated. #p < 0.05 comparing doses).

To determine sensitivity to DFMO treatment, cell viability was measured using the calcein AM cell viability assay. BE(2)-C, SMS-KCNR, and CHLA90 cell lines were treated for 72 hours with DFMO concentrations ranging from 0.156 mM to 80 mM (Figure 2). The BE(2)-C cell line was the most sensitive to DFMO treatment with an IC_50_ of 3.0 mM, followed by the SMS-KCNR cell line which had an IC_50_ of 10.6 mM. The CHLA90 cell line was the least sensitive with an IC_50_ of 25.8 mM. These data confirm that sensitivity to DFMO correlates with higher *LIN28B* and *MYCN* gene expression in all three cell lines (BE(2)C>SMS KCNR>CHLA90). Sensitivity to DFMO varied between cell lines in other *in vitro* experiments as well. Compared to BE(2)-C and SMS-KCNR cell lines, CHLA90 cells required a longer treatment time to show decreases in LIN28B and MYCN protein expression when treated with DFMO (Figure 3A). Additionally, the CHLA90 cell line did not exhibit any increase in Let-7 miRNA expression after DFMO treatment for 6 hours (Figure 3B) or a decrease in ATP/cell levels after DFMO treatment for 72 or 96 hours (Figure 5). Significant increase in Let-7 expression and decrease in ATP/cell were seen in both BE(2)-C and SMS-KCNR cell lines.

### DFMO treatment reduces LIN28B and MYCN protein expression in NB cell lines

Western blot analysis was used to show that LIN28B and MYCN protein levels decreased with DFMO treatment (Figure 3A). BE(2)-C, SMS-KCNR, and CHLA90 cell lines were treated for 48, 72, or 96 hours with 5 mM or 10 mM DFMO. For BE(2)-C and SMS-KCNR cell lines, a decrease in LIN28B protein could be seen as early as 48 hours with 5 mM and 10 mM DFMO treatment and even greater effects were seen after 72 and 96 hours. In CHLA90 cells a small decrease in LIN28B protein expression was evident at 72 hours with both 5 mM and 10 mM DFMO treatment and cells exhibited a substantial decrease in LIN28B protein at 96 hours. In BE(2)-C and SMS-KCNR cell lines, MYCN protein expression followed the same trend as LIN28B protein expression, decreasing at after 48, 72, and 96 hours with both 5 mM and 10 mM doses of DFMO. For the CHLA90 cell line, MYCN protein expression was unchanged at all three timepoints with both 5 mM and 10 mM doses of DFMO.

### DFMO treatment affects Let-7 miRNA expression in NB cell lines

qRT-PCR analysis demonstrated changes in Let-7 miRNA expression in response to DFMO treatment (Figure 3B). BE(2)-C, SMS-KCNR, and CHLA90 cell lines were treated for 6 hours with 5 mM or 10 mM DFMO. Significant increases in Let-7 miRNA expression were evident for both BE(2)-C and SMS-KCNR cell lines with both 5 mM and 10 mM DFMO treatments when compared to untreated cells. BE(2)-C cells exhibited a 1.4 fold increase in Let-7 miRNA expression with 5 mM DFMO treatment (*p* < 0.05 and a 3.6-fold increase with 10 mM treatment (*p* < 0.001). SMS-KCNR cells showed a 2.4 fold increase in Let-7 miRNA expression with 5 mM treatment (*p* < 0.05) and 2.5 fold increase with 10 mM treatment (*p* < 0.01). In the BE(2)-C cell line10 mM treatment increased Let-7 miRNA expression an additional 2.2 fold when compared to the 5 mM treatment group (*p* < 0.01), indicating a significant dose effect. No significant dose effect was seen in the SMS-KCNR cell line. For the CHLA90 cell line, there was a significant decrease in Let-7 miRNA expression with both 5 mM (*p* < 0.01) and 10 mM (*p* < 0.001) treatments. These results demonstrate that DFMO treatment does affect Let-7 miRNA levels in NB cells lines and that these effects are most pronounced in cells with high LIN28B and MYCN gene expression.

### DFMO inhibits neurosphere formation

**Figure 4:**
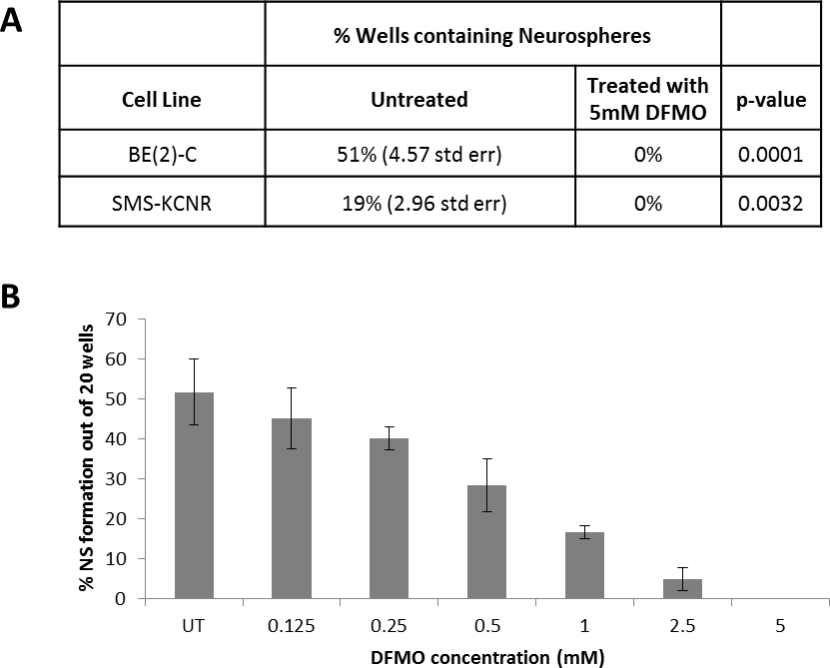
Neurosphere Formation Assay A) Both BE(2)-C and SMS KCNR cell lines show inhibition of neurosphere formation in the presence of 5 mM DFMO. B) Treatment with DFMO shows a dose dependent decrease in neurosphere formation in BE(2)-C cells (n=3 separate experiments).

The neurosphere assay is one of the most frequently used methods to isolate, expand and also calculate the frequency of neural stem cells. Neurosphere assays were performed using BE(2)-C cells which showed a dose dependent decrease in neurosphere formation with DFMO treatment. At 5 mM DFMO all neurosphere formation was inhibited in triplicate assays (p < 0.01) for both SMS-KCNR and BE(2)-C cell lines. With the addition of increasing DFMO concentrations, there was a 9-fold decrease in percentage of neurospheres formed between 0.125 and 2.5 mM (Figure 4).

### DFMO treatment decreases ATP levels in NB cell lines

**Figure 5:**
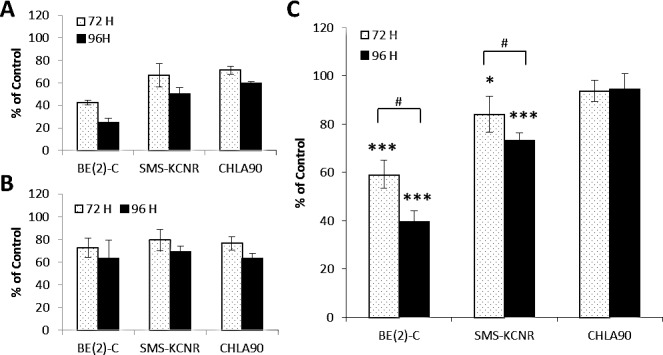
ATP analysis for NB cells treated with 10 mM DFMO for 72 or 96 hours All data are presented as percent of untreated control. A) Total ATP per sample treated with 10 mM DFMO. B) Total cell number per sample treated with 10 mM DFMO. C) Ratio of total ATP over total cell number (ATP/cell). BE(2)-C cells had the greatest decrease in ATP/cell with DFMO treatment with a 40.8% decrease in ATP/cell at 72 hours and a 60.5% decrease at 96 hours when compared to untreated. SMS-KCNR cells also had decreased ATP/cell after treatment with DFMO at 72 (15.5% decrease) and 96 hours (26.9% decrease). There were no significant change in ATP/cell levels in CHLA90 cells after DFMO treatment at either timepoint. *p < 0.05, ***p < 0.001 relative to untreated. #p < 0.05 comparing timepoints.

To quantify the effect of DFMO treatment on glycolytic metabolism in NB cell lines ATP levels in NB cells were measured by combining assays that measure ATP levels and cell number (ATP/cell). ATP/cell assays were performed in BE(2)-C, SMS-KCNR, and CHLA90 cells after 72 or 96 hours of treatment with 10 mM DFMO (Figure 5). Total ATP was decreased in all cell lines at both timepoints when compared to untreated (Figure 5A). Cell number was also decreased in all samples when compared to untreated (Figure 5B); however, there were no significant differences in cell number between cell lines. The ratio of ATP/cell was significantly lower in the BE(2)-C cell line after treatment with DFMO for both 72 hours (*p* < 0.001) and 96 hours (*p* < 0.001). ATP/cell also decreased significantly in SMS-KCNR cells for both 72 hour (*p* < 0.05) and 96 hour (*p* < 0.001) timepoints. ATP/cell was significantly lower at 96 hours compared to 72 hours for both BE(2)-C (*p* < 0.01) and SMS-KCNR (*p* < 0.05) cell lines, indicating that longer exposure to DFMO treatment is more effective. CHLA90 cells exhibited no significant changes in ATP/cell after 72 or 96 hours of treatment with DFMO. These results indicate that DFMO treatment has significant effects on glycolytic metabolism in NB cells, especially in cell lines with high LIN28B and MYCN gene expression.

### DFMO treatment reduces glycolytic metabolism *in vivo*

**Figure 6:**
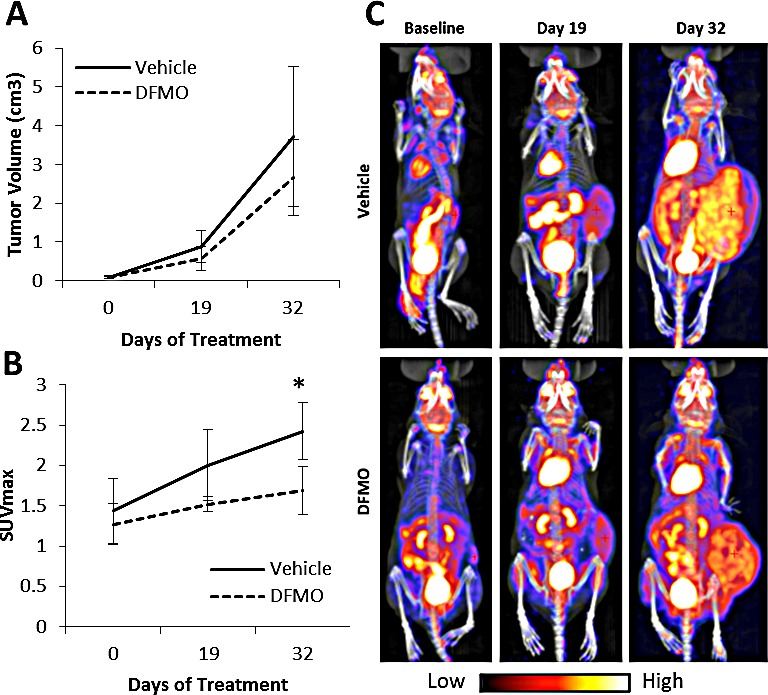
DFMO treatment *in vivo* Mice began treatment with 2% DFMO in drinking water after tumors reached approximately 0.2 cm^3^. PET/CT scans were done at baseline, 19 days, and 32 days after treatment was initiated. A) Average NB tumor volume was quantified using CT scans. There was no significant difference in tumor volume between vehicle and DFMO treatment groups. B) Average tumor SUVmax was quantified using PET scans. Average SUVmax was lower in mice treated with DFMO at 19 days and 32 days of treatment when compared to vehicle. C) PET images of one vehicle treated mouse and one DFMO treated mouse at all three timepoints. The DFMO treated mouse showed less ^18^F-FDG uptake in the tumor at both 19 days and 32 day days when compared to the vehicle (*p < 0.05).

*In vivo* studies demonstrated that DFMO treatment reduced glycolytic metabolic activity as measured by a decrease in PET activity in tumors measured using SUVmax in DFMO-treated mice (Figure 6). SUVmax was considerably lower in DFMO-treated mice after 19 days of treatment compared to untreated mice, though results were not significant (*p* < 0.08). SUVmax was significantly lower for DFMO-treated mice after 32 days of treatment compared to untreated mice (*p* < 0.05). Tumor volume was approximately 1.0 cm^3^ lower in the DFMO-treated group after 32 days when compared to untreated. No DFMO treated mice exhibited any signs of DFMO toxicity (n = 4 mice per group).

## DISCUSSION

Our study is the first to demonstrate that DFMO treatment restores balance to the LIN28/Let-7 axis and inhibits glycolytic metabolism in NB. This is demonstrated by the decrease in LIN28B protein levels (Figure 3A) and increase in Let-7 miRNA expression (Figure 3B) seen with DFMO treatment. This change in the LIN28/Let-7 axis also appears to affect MYCN protein expression since MYCN is seen to decrease in the two cell lines tested which overexpress MYCN (Figure 3A). An interesting finding was that higher LIN28B and MYCN gene expression levels correlate with greater sensitivity to DFMO in NB cell lines. BE(2)-C cells, which have high LIN28B and MYCN expression (Table 1), were the most sensitive to DFMO treatment in all *in vitro* experiments (Figures 2-5). Conversely, CHLA90 cells had low expression for both LIN28B and MYCN (Table 1) and were consistently the most resistant to DFMO treatment (Figures 2, 3, 5). The lack of change in MYCN protein expression seen in the CHLA90 cell line was expected since CHLA90 cells have baseline low MYCN gene expression. SMS-KCNR cells had high MYCN and moderate LIN28B expression relative to BE(2)-C and CHLA90 cells (Table 1) and had consistent moderate sensitivity to DFMO treatment (Figures 2-5). These data are consistent with other studies showing that DFMO treatment affected LIN28/Let-7 in colorectal cancer [13] and that changes in MYCN protein expression were seen with changes in the LIN28/Let-7 axis in other studies of NB at these doses [21]. These changes occur at doses correlating to established IC50's within range of those previously reported in NB [4, 8]. These results reinforce claims that polyamines play an important role in the LIN28/Let-7 pathway [13], since those cell lines with the greatest LIN28B and MYCN gene expression were most sensitive to a treatment that depletes polyamine levels. The findings also indicate that DFMO treatment may be more effective for NB patients with overexpression of genes that play a role in the LIN28/Let-7 axis. As such, genomic analysis of patient tumors may be beneficial in predicting response to DFMO therapy.

Changes in bioenergetics of NB cell lines treated with DFMO, as measured by ATP/cell, suggest that DFMO treatment may result in a reduction of glycolytic metabolism in NB (Figure 5). Many cancer cells are reprogramed to produce ATP primarily via glycolytic metabolism as opposed to oxidative phosphorylation, a phenomenon termed the “Warburg effect” [24, 30-35]. Because this phenomenon is well documented in NB [36], we used ATP/cell as a measure of glycolytic metabolic activity in NB. BE(2)-C and SMS-KCNR cell lines had decreased ATP/cell levels when treated with DFMO. These data indicate that DFMO treatment reduces metabolic activity in these cell lines. This reduction in glycolytic metabolism can be attributed to the restoration of balance in the LIN28/Let-7 axis caused by DFMO treatment. A recent study showed that changes in the LIN28/Let-7 signaling pathway led to changes in glycolytic metabolism in NB [22]. In this study, higher levels of LIN28 protein correlated with greater phosphorylation of AKT and S6, both of which play important roles in the insulin-PI3K-mTOR signaling pathway that regulates glycolytic metabolism. This indicates that the LIN28/Let-7 axis plays a significant role in regulating glycolytic metabolic activity in NB.

To further validate that DFMO treatment reduces glycolytic metabolism in NB, we tested the effects of DFMO treatment on ^18^F-FDG uptake of NB tumors through an *in vivo* microPET study (Figure 6). It has been proven that PET scans provide a quantifiable measure of glycolytic metabolism in living tumors by measuring the breakdown of ^18^F-FDG (^18^F radiolabeled glucose analogue) [37]. It has been previously established that administration of 2% DFMO in drinking water resulted in optimal reduction in both tumor cell polyamine levels and tumor size in melanoma models [38-41]. In our model, there was a significant reduction in ^18^F-FDG uptake in these tumors as well. These data, along with the PET scans from patients enrolled in the phase I DFMO clinical trial [9], indicate that DFMO treatment has a substantial impact on glycolytic metabolism in tumors. This study also suggests that because tumor activity changed with little change to tumor volume, ^18^F-FDG PET imaging may be a tool to consider in the evaluation of the therapeutic response to DFMO treatment in both pre-clinical and clinical settings.

Interestingly, recent studies have shown that *LIN28* is an important oncogenic driver in cancer stem cells [15, 42] which may evade standard chemotherapeutic approaches and cause relapse in patients. Additionally, studies have shown that cancer stem cells depend on glycolytic metabolism for ATP production [43]. Neurosphere formation requires the presence of cancer stem cells within wells. In the presence of DFMO, inhibition of neurosphere formation suggests that DFMO has affected the stem cells ability to form neurospheres. Since our findings show that DFMO affects these important cancer stem cell pathways and inhibits neurosphere formation in NB, we will further elucidate the effects of DFMO on NB cancer stem cells in future studies.

Overall, our work shows that inhibiting polyamine biosynthesis with DFMO treatment effectively restores balance to the LIN28/Let-7 axis. These results have exciting clinical implications. The reduction in glycolytic metabolism via DFMO treatment holds promise for reducing tumor metabolic activity in NB. Additionally, cells with higher *LIN28B* and *MYCN* gene expression are more sensitive to DFMO treatment, so DFMO may prove an effective therapy for aggressive, *MYCN*-amplified NB. Finally, these results provide a new perspective on the use of ^18^F-FDG PET imaging in pre-clinical and clinical DFMO studies. Since tumor activity may change significantly with little or no change to tumor volume, ^18^F-FDG PET may offer more meaningful information in DFMO studies than other imaging tools, both for planning treatment and monitoring disease progression.

## MATERIALS AND METHODS

### Inhibitor

The ODC inhibitor DFMO (kindly provided by Dr. Patrick Woster, Medical University of South Carolina) was dissolved at 1 M in H_2_O. Drug stocks were stored in small aliquots at −20°C and diluted with culture media on the day of the experiment.

### Cell lines and cell culture

Drug testing was conducted *in vitro* on three human NB cell lines: BE(2)-C (purchased in 2011 from ATCC, **Manassas, VA**), SMS-KCNR (kindly donated by Dr. John Maris in 2004, The Children's Hospital of Philadelphia, PA), and CHLA90 (kindly donated by Patrick Reynolds in 2006**, The Children's Hospital of Los Angeles, CA**). SMS-KCNR cells were also used for drug testing *in vivo.* Two of the cell lines tested were MYCN amplified (BE(2)-C and SMS-KCNR) and one was non-amplified (CHLA90). All cell lines were validated by comparing STRs in May 2012 (DNA Diagnostics Center, Fairfield, OH). Cells were cultured in RPMI-1640 with 10% (vol/vol) certified fetal bovine serum (FBS), 100 U/mL penicillin, and 100 mg/mL streptomycin. Incubation was at 37°C with 5% CO_2_. All cell culture reagents were purchased from Life Technologies (Grand Island, NY). Cells were grown in 6-well or 96-well plates and were allowed to grow to 60-70% confluence prior to addition of drugs.

### RNA expression profiling analysis

RNA expression analysis was performed for untreated BE(2)-C, SMS-KCNR, and CHLA90 cells. 5 × 10^4^ cells were collected into 1.5 mL eppendorf tubes and centrifuged at 14,000 rpm for 10 minutes at 4°C to pellet. Pellets were rinsed twice with 500 μL of ice cold PBS with 1 μL SuperaseIn RNase inhibitor (Ambion, Austin, TX) before adding 250 μl of ice cold PBS + 2 μL SuperaseIn (Ambion) and storing at −80°C. All samples were shipped to Clinical Research Laboratories (CRL) (Lenexa, KS) where Affymetrix GeneChip U133 Plus 2.0 genome wide expression cDNA microarray was used to determine RNA expression. All cell lines were tested in duplicate. All analysis was done using R/Bioconductor packages and Partek Genomics Suite.

### Cell viability and IC_50_ values

Calcein AM fluorescent assay (Molecular Probes, Eugene, OR) was used to determine cell viability in 96-well plates. SMS-KCNR cells were plated at 5,000 cells/well, CHLA90 cells were plated at 4,000 cells/well, and BE(2)-C cells were plated at 3,000 cells/well. Cells were then treated with increasing concentrations (0.156-40 mM) of DFMO in RPMI-1640 with 10% FBS for 72 hrs. After incubation with drug for 72 hrs, the medium was aspirated and replaced with phenol-red free RPMI medium containing 2 μg/mL Calcein AM. After 30 minutes incubation at 37°C, fluorescence was measured at 480ex/520em with a BioTek plate reader (BioTek Instruments Inc, Winooski, VT). IC_50_ values were calculated with a four-parameter variable-slope dose response curve using GraphPad Prism v.5 software.

### Western Blotting

Cells were plated at 100,000 cells/well in 6-well plates and were allowed to adhere overnight prior to treating with 5 mM DFMO in RPMI-1640 with 10% FBS for 48, 72, or 96 hours. Drug supplemented medium was refreshed after 48 hours for 72 and 96 hours timepoints. H_2_O was used as vehicle control. Cells were lysed with RIPA lysis buffer (Cell Signaling, Danvers, MA), supplemented with a complete protease inhibitor (Roche, Madison, WI). Cell lysates were collected and frozen at −80°C overnight to ensure complete cell lysis. Protein concentrations were determined by Bradford assay (Bio-Rad, Hercules, CA). Lysates were electrophoresed on a 12% SDS-polyacrylamide gel in running buffer (Bio-Rad). Thirty μg of protein was loaded per lane. Gels were semi-dry transferred to nitrocellulose membranes using the Turbo Transfer system with Turbo Transfer buffer (Bio-Rad). Primary antibodies used were: rabbit polyclonal LIN28B, rabbit polyclonal MYCN, and rabbit polyclonal β-actin (Cell Signaling). Goat anti-rabbit secondary antibody was used (Licor). Protein bands were detected by fluorescence using the Odyssey infrared imaging system (Licor).

### Quantitative RT-PCR

qRT-PCR analysis was used to evaluate Let-7 miRNA expression. Cells were plated at 100,000 cells/well in 6-well plates and allowed to adhere overnight prior to drugging with 5 mM or 10 mM DFMO for 6 hours. Lysates were collected using Trizol (Invitrogen, Carlsbad, CA) then stored in −80°C for up to 1 month prior to RNA extraction. RNA was extracted using BCP (Sigma, St. Louis, MO) and RNA cleanup was performed using the miRNeasy Mini Kit (Qiagen, Valenca, CA). cDNA was made using the TaqMan MicroRNA Reverse Transcription Kit (Applied Biosystems, Foster City, CA) along with RT primers specific for human Let-7i-5p or RNU44 (Applied Biosystems). All sets of reactions were run in triplicate using 30-100 ng cDNA per reaction, Taqman Fast Advanced Master Mix and Let-7i-5p and RNU44 specific primers (Applied Biosystems). Quantitation was performed using ΔΔC_T_; each treatment was normalized to the untreated sample.

### Neurosphere Assays

Neuroblastoma cell lines BE(2)-C and SMS-KCNR cells were plated in RPMI media containing 10% FBS, 1% Pen/Strep at a concentration of 2 cells/well in ultra-nonadherent 96-well plates. (Corning cat#3474) The final cell concentration was 40 cells/mL, with 50 μl added to the inner 60 wells of each plate. 24 hours after plating, a serial dilution of DFMO was added for final drug concentrations of 5, 2.5, 1, 0.5, 0.25, 0.125, and 0 mM. DFMO-containing media was refreshed weekly. On days 5 and 10 after drug treatment, all wells were scanned under 10X light microscopy and the number of neurosphere per well documented. Neurospheres were monitored until day 20 of treatment for changes and growth.

### ATP per cell

The Cell Titer GLO luminescent assay (Promega, Madison, WI), which measures total ATP levels, was combined with the CyQuant fluorescent DNA assay (Invitrogen, Grand Island, NY) to measure ATP level per cell. SMS-KCNR cells were plated at 5,000 cells/well, CHLA90 cells were plated at 4,000 cells/well and BE(2)-C cells were plated at 3,000 cells/well in 96-well black-walled plates. Cells were treated for 72 or 96 hrs with 5 mM or 10 mM DFMO in RPMI-1640 with 10% FBS. 1% H_2_O in RPMI-1640 was used as control. After 72 or 96 hrs, medium was aspirated and replaced with Cell Titer GLO reagent (lysis buffer mixed with Cell Titer GLO substrate, diluted with PBS 1:1) supplemented with CyQuant stock solution (50 μL dye per 10 mL Cell Titer GLO reagent). Plates were rocked on an orbital shaker for 3 minutes at 20°C to induce cell lysis, and then were incubated an additional 10 minutes at 20°C to allow luminescent signal stabilization. Luminescence and fluorescence data were recorded using a Wallace plate reader and Envision software. Cell Titer GLO data were divided by CyQuant data and normalized to vehicle wells in order to generate data on ATP per cell.

### *In vivo* Studies

*In vivo* drug studies were conducted with four-week-old female nude mice (nu/nu) from Charles River Laboratories (Portage, MI). Mice were housed in pathogen-free conditions and cared for in accordance with the International Animal Care and Use Committee (IACUC) standards. Mice were injected with 10^7^ SMS-KCNR cells suspended in 100 μL of Matrigel (BD Biosciences, San Jose, CA). Tumor cells were injected subcutaneously into the right flank. Tumors were allowed to grow to approximately 0.3 cm^3^ before separating mice into untreated and DFMO-treated groups with the same average tumor volume. Both groups underwent baseline PET/CT scans prior to starting drug treatment. 2% DFMO was administered in drinking water (4 g DFMO into 200 mL H_2_O). Water was changed twice weekly. All mice underwent PET/CT scans when average tumor volume reached 1.0 cm^3^ and again at 2.5 cm^3^. Caliper tumor measurements were completed bi-weekly until tumors reached a volume of 1.5 cm^3^ at which point tumors were measured 3 times per week. Throughout the study average weight for mice in both treatment groups was approximately 20 g. All mice were euthanized when the first mouse reached a tumor volume of 3.0 cm^3^.

### PET/CT Scans

All mice were fasted with free access to water for at least 6 hours prior to 2′-deoxy-2′-^18^F-fluoro-D-glucose (^18^F-FDG) injection. Mice were placed in a 37.5 °C heated cage 20-30 minutes prior to radiotracer injection and moved to a 37.5 °C heated induction chamber 10 minutes prior to injection where they were anesthetized with 2% isoflurane in 1000 cc/min O^2^. A dose of 25 μCi in 0.1 ml of ^18^F-FDG was administered by tail vein injection and the mice were placed in a 37.5 °C heated induction chamber for 60 minutes of unconscious uptake. PET scans were acquired using a Genisys^4^ PET (Sofie Biosciences, Culver City, CA) system and CT scans were acquired using a Bioscan NanoSPECT/CT (Washington, D.C.). A 10 minute static acquisition was used for PET imaging followed immediately by a 6.5 minute CT acquisition both utilizing the mouse imaging chamber from the Genisys^4^. PET reconstruction was performed without attenuation correction using 3D Maximum Likelihood Expectation Maximization with 60 iterations and CT reconstruction used Filtered Back Projection with a Shepp-Logan Filter. PET and CT reconstructions were exported in dicom image format, fused and analyzed using custom software developed by the Small Animal Imaging Facility at Van Andel Institute. Standardized Uptake Values were calculated using the mouse body weight and corrected for residual dose in the injection syringe and the injection site, as applicable. The formula used to calculate SUV was $\mathrm{SUV}=\frac{{\text{Activity}}_{\text{tissue}}/{\text{Volume}}_{\text{tissue}}}{\text{Injected Activity}/\text{BodyWeight}}$. Maximum Standardized Uptake Value (SUVmax) was used to evaluate drug response in this study.

## Abbreviations

NB: Neuroblastoma
ODC: Ornithine Decarboxylase
DFMO: Difluoromethylornithine
PET: Positron Emission Tomography
18F-FDG: 2′-deoxy-2′-18F-fluoro-D-glucose
SUVmax: Maximum Standard Uptake Value
